# Application of Hyphenated Techniques in Speciation Analysis of Arsenic, Antimony, and Thallium

**DOI:** 10.1100/2012/902464

**Published:** 2012-05-02

**Authors:** Rajmund Michalski, Sebastian Szopa, Magdalena Jabłońska, Aleksandra Łyko

**Affiliations:** Institute of Environmental Engineering, the Polish Academy of Sciences, 34 Skłodowskiej-Curie Street, 41 819 Zabrze, Poland

## Abstract

Due to the fact that metals and metalloids have a strong impact on the environment, the methods of their determination and speciation have received special attention in recent years. Arsenic, antimony, and thallium are important examples of such toxic elements. Their speciation is especially important in the environmental and biomedical fields because of their toxicity, bioavailability, and reactivity. Recently, speciation analytics has been playing a unique role in the studies of biogeochemical cycles of chemical compounds, determination of toxicity and ecotoxicity of selected elements, quality control of food products, control of medicines and pharmaceutical products, technological process control, research on the impact of technological installation on the environment, examination of occupational exposure, and clinical analysis. Conventional methods are usually labor intensive, time consuming, and susceptible to interferences. The hyphenated techniques, in which separation method is coupled with multidimensional detectors, have become useful alternatives. The main advantages of those techniques consist in extremely low detection and quantification limits, insignificant interference, influence as well as high precision and repeatability of the determinations. In view of their importance, the present work overviews and discusses different hyphenated techniques used for arsenic, antimony, and thallium species analysis, in different clinical, environmental and food matrices.

## 1. Speciation Analytics and Hyphenated Techniques

Speciation, as word borrowed from biology, is a term describing the existence of various chemical and physical forms of a particular element while speciation analytics denotes the determination of those forms [1]. The notion of speciation is used in chemistry to determine the occurrence of diverse forms of a given element (e.g., element at various oxidation states or bound with different ligands) in the analysed sample. The forms might differ in physical and chemical characteristics as well as in the influence they exert on living organisms. In the last several decades, speciation analytics has become one of the most central issues in analytical chemistry [2]. 

Even though its cost is significant, speciation analytics has been gaining greater importance when it comes to solving problems that concern not only the determination of total element contents but also taking into account various forms of occurrence. It plays an exceptional role in the examination of biochemical cycles of selected chemical compounds, determination of toxicity and ecotoxicity of selected elements, food and pharmaceutical product quality control, technological process control, as well as health risk assessment and clinical analytics [3]. Within the speciation analytics framework, determination of substances produced and emitted into the environment by humans and the analysis of natural compounds (formed as a result of biochemical transformations in living organisms or in the environment) can be differentiated. Environmental analytics deals with the former group while the latter one involves the biochemical and ecotoxic examinations.

It is also reasonable to differentiate between chemical and physical speciation. It is possible to distinguish between screening speciation (searching for and determining selected chemical forms) and distribution speciation (searching for and determining selected chemical individuals in specific elements of the examined sample) in chemical speciation. Another division within chemical speciation concerns group speciation (searching for and determining specific groups or classes of chemical forms) and individual speciation (searching for and determining all chemical individuals present in the sample) [4]. When it comes to liquid sample analyses, the most common technique is the one developed by Florence and Batley [5].

According to this method, a water or wastewater sample filtered through a 0.45 *μ*m filter is divided into a solid phase and mobile one, in which the determinations of total metal contents as well as the metal labile and bound forms are carried out.

The division suggested by Tessier et al. [6] is recommended in the research concerning the speciation of heavy metals in bottom sediments. They distinguished and defined five fractions, that is, exchangeable metals, carbonate-bound metals, iron and manganese oxides-bound metals, organic matter-bound metals, and other mineral-bound metals. Nonetheless, this method of speciation does not allow the differentiation between oxidation states of elements, which may be of great importance when considering their toxicity.

The analyte determination completes the procedure in which sampling is one of the most crucial stages. It is particularly important in speciation analytics as even the routine processes, such as dilution, pH changes caused by sample preservation, temperature, and pressure alternations, can bring about irreversible transformations in the primary analyte form [7]. Particular difficulties appear when the sample is collected under conditions considerably dissimilar to those in which it is later analyzed. It happens for instance when samples are collected from the lower strata of water bodies. Pressure drops trigger the emission of gaseous elements. For example, if it is CO_2_, then the increase of the sample pH, acid-base balance changing, and increasing stability of complexes as well as precipitation of sparingly soluble sediments follow. 

The lability and changeability of the sample is crucial when biological material is studied. Microbiological, enzymatic, photochemical, and other processes whose character is often unclear and unexpected can still occur in these samples after their collection [8]. Lowering the detection limits of analytes to extremely low concentration levels resulted in the fact that methods used so far did not always meet the necessary requirements [9]. For that reason, there has been a tendency for several years to combine various methods and techniques. These combinations are known as the hyphenated techniques. A suitable hyphenated technique should be selective towards determined analytes, sensitive within a wide range of concentrations, and ought to enable possibly best identification of the determined substances.

In speciation analytics, chromatographic methods are largely used for separation [10] whereas spectroscopic ones are employed for detection [11, 12], albeit the application of other methods is also possible [13]. The application of hyphenated techniques entails perfect understanding of analytical methodologies and detailed knowledge of the apparatus. These are expensive systems used in scientific research rather than in routine analyses. 

The earliest hyphenated techniques were developed by coupling gas chromatography with various detectors. The following systems were elaborated: GC-AAS (Gas Chromatography—Atomic Absorption Spectrometry), GC-AES (Gas Chromatography—Emission Atomic Spectrometry), GC-MS (Gas Chromatography—Mass Spectrometry) or GC-ICP-MS-TOF (Gas Chromatography—Inductively Coupled Plasma—Mass Spectrometry—Time of Flight Mass Spectrometry). 

Due to technological reasons, systems employing liquid chromatography methods for separation of analyzed substances, such as HPLC-ICP-MS (High Performance Liquid Chromatography-Inductively Coupled Plasma—Mass Spectrometry), appeared in the market slightly later. It is often thought that there exist elements (or their specific chemical forms) essential for the proper functioning of living organisms and elements (or their compounds) that interfere with metabolic processes. To paraphrase Paracelsus, a physician and chemist living in the 16th century, what ought to be discussed are significant or toxic concentrations or the amounts of elements and their diverse chemical forms. 

The results of toxicological tests indicate that in many cases it is not the total content of a given element but the share of its particular forms that has a decisive influence on living organisms. Due to that fact, the knowledge of various elemental forms is more important than the information on its total content [14]. Elements occurring in ionic forms predominantly demonstrate biological activity and toxicity affecting living organisms. Ion chromatography is the most popular method serving to separate and determine organic and inorganic ionic substances [15]. It is applied in the hyphenated techniques and speciation analytics mainly for the determination of selected water disinfection by-products [16], as well as ions and metalloids [9].

Among the most popular hyphenated techniques used to determine different ionic forms of metals and metalloids, there are couplings of various liquid chromatography types. These include HPLC (High Performance Liquid Chromatography), IC (Ion Chromatography), I-EC (Ion-Exclusion Chromatography), or SEC (Size Exclusion Chromatography) with ICP MS (Inductively Coupled Plasma Mass Spectrometry) or ESI MS (Electrospray Ionization Mass Spectrometry) [17]. The most popular hyphenated techniques utilizing ion chromatography are IC-ICP-MS (Ion Chromatography—Inductively Coupled Plasma—Mass Spectrometry), IC-ICP-OES (Ion Chromatography-Inductively Coupled Plasma-Optical Emission Spectrometry), and IC-MS (Ion Chromatography—Mass Spectrometry) [18, 19].

The application of mass spectrometry detection allows not only to obtain the information on the qualitive and quantitative content of the sample but also to determine the structure and molar masses of the analytes. The main difficulties in using mass spectrometry detector coupled with chromatographic methods result from the fact that it is necessary to maintain very low pressure in the spectrometer while separated analyte ions leave the chromatographic column under comparatively high pressure. 

While it was relatively easy to couple a gas chromatograph with mass spectrometry detector, a large amount of the eluate in the case of liquid chromatography was a major obstacle in the introduction of HPLC-ESI-MS system into the laboratory practice [20]. Various ionization sources can be utilized in the HPLC-ESI-MS apparatus. These include ESI (Electrospray Ionization), APCI (Atmospheric Pressure Chemical Ionization), or APPI (Atmospheric Pressure Photochemical Ionization). The range of these applications depends on the polarity and mass of the analytes as well as the eluent flow rate. MS detection can be carried out in SIM (Selected Ion Monitoring) or SM (Scan Mode) modes. The former provides the information on the analyte mass while the latter offers data regarding mass spectra and mass distribution. The problems concerning identification in the case of large molecules relate mainly to the fact that there is a higher number of possibilities that the obtained spectra will have the same ratios of mass to charge.

It is estimated that approxmately 50% of all publications on the subject of speciation analytics involve only five elements, that is, arsenic, selenium, mercury, chromium, and tin.

Further 30% of papers are devoted to copper, zinc, lead, and iron [21]. The following literature review discusses arsenic, antimony, and thallium. The described elements have complex physical and chemical characteristics and are of great interest for both toxicologists and analytical chemists. Among them, arsenic and its compounds are the best known and described. Less information on antimony is available while thallium and it compounds are still the most mysterious and unfamiliar.

## 2. Arsenic

Arsenic is a metalloid from group 15 of the periodic table. Its most important oxidation states are of −3, +3, and +5. Arsenic compounds were known in the Antiquity. Later, it gained great importance in medicine; it is even said to have been the basis of the modern pharmacology. People started using organoarsenic compounds at the turn of the nineteenth and twentieth centuries.

They turned out to be far less toxic for humans and animals than the inorganic ones. Nevertheless, nearly all arsenic medicines were withdrawn from the market in the second part of the previous century. Even though they were highly effective, they were also carcinogenic. Nowadays, arsenic compounds are used in medicine only very seldom. However, the interest in employing arsenic trioxide in the antineoplastic therapy has increased in recent years [72].

Arsenic has also been used in semiconductor production (as gallium arsenide), improving the quality of selected alloys, chemical warfare weapon industry, and wood conservation. It has also been added to glass as it provides a greenish glow. In addition, arsenic compounds have been employed in tanning and as pigments and pesticides [73]. Arsenic occurs in several hundred minerals, mainly in pyrite, and in lead and copper ores. It also appears in nature in a number of organic compounds such as arsenate (arsenic acid)—H_3_AsO_4_, arsenite (arsenious acid)—HAsO_2_, dimethylarsonic acid DMAA—(CH_3_)_2_AsO(OH), cacodylic acid (CH_3_)_2_AsO_2_H, dimethylarsine DMA—(CH_3_)_2_AsH, methylarsine, trimethylarsine oxide TMAO—(CH_3_)_3_AsO, tetramethylarsonium salts, arsenobetaine AsB—(CH_3_)_3_As^+^CH_2_COOH and arsenocholine AsC—(CH_3_)_3_As^+^CH_2_ CH_2_OH.

Anthropogenic sources of arsenic and its compounds comprise by-product emissions in mining and smelting of nonferrous metal ores and fossil fuels combustion (mainly brown and hard coals). Total world production of arsenic amounted to approximately 75,000 tonnes in 2005. Around three quarters of this amount was used for wood preservation and one fifth in agriculture for herbicide manufacturing. The remaining amount was employed in nonferrous alloys and glass production. Whereas in highly industrialized countries applications of arsenic and its compounds have become more and more limited, they are still broadly used in developed countries, even though their toxic characteristics are widely known. It is estimated that 20% of population in Bangladesh consume water highly contaminated with arsenic and its compounds [36]. Arsenic is a very mobile element. For that reason, it occurs in all units of the environment. It easily migrates from the lithosphere into the hydrosphere, and its content in natural water is highly diversified and determined by the ground type and water pollution. Regulations that are in force in most European countries state that the total arsenic content in soil ought not to exceed 20 mg/1 kg of soil. Its admissible amount in drinking water amounts to 10 *μ*g/L, although its concentrations can exceed even several dozen mg/L in surface and underground water depending on geological conditions.

In the atmosphere, arsenic occurs mainly in the forms of AsO_3_ and volatile organic compounds. Its mean concentration ranges between 1 ng/m^3^ in the countryside, 2 ng/m^3^ in the urban areas, and up to 50 ng/m^3^ in industrial regions.

Selected arsenic compounds determined with its speciation analytics encompass:

- inorganic ones such as arsenite (arsenious acid)—HAsO_2_—and arsenate (arsenic acid)—H_3_AsO_4_ among the inorganic compounds, H_2_AsO_4_^−^ (pH = 2–7), HAsO_4_^2−^ (pH > 7), and H_3_AsO_3_ (pH<9) are stable;
- organic ones such as monomethylarsine MMA—CH_3_AsH_2_, monomethylarsonic acid MMAA—CH_3_AsO(OH)_2_, dimethylarsine DMA—(CH_3_)_2_AsH, dimethylarsonic acid DMAA—(CH_3_)_2_AsO(OH), arsenobetaine AsB—(CH_3_)_3_As^+^CH_2_COOH, arsenocholine AsC—(CH_3_)_3_As^+^CH_2_CH_2_OH, trimethylarsine oxide TMAO—(CH_3_)_3_AsO, tetramethylarsonium ion Me_4_As^+^—(CH_3_)_4_As^+^, and arsenic-containing ribosides arsenosugars—various sugar structures [117].

All arsenic compounds, to a higher or lesser extent, possess carcinogenic and protoplasmatic (they destroy bacteria cell walls) characteristics. However, the toxicity of arsenic compounds depends on the form in which they are consumed and on their mobility. In general, it is assumed that their harmfulness decreases in the following order: arsines > inorganic arsenites > organic trivalent compounds (arsenoxides), inorganic arsenates > organic pentavalent compounds > arsonium compounds > elemental arsenic. Arsenobetaine and arsenocholine are considered nontoxic. The symptoms of the chronic poisoning appear usually after a few years. They may include various forms of kidney, lung, liver, skin, or urinary bladder neoplasms. A prolonged skin contact with the arsenic dust can cause several types of skin cancer. What is interesting, long-term ingestion of low doses of arsenic compounds increases the body resistance against acute poisoning. In December 2010, there appeared information about the discovery of arsenophile GFAJ-1 bacteria that were able to sustain its growth in culture medium in which phosphorous had been replaced with arsenic (http://www.nasa.gov/topics/universe/features/astrobiology_toxic_chemical.html).

It was suggested that arsenic might have been incorporated into biomolecules (e.g., DNA) which maintained its proper biological activity in this form. Nevertheless, this information was denied in March 2011. Arsenic speciation analytics makes use of the hyphenated techniques, such as HPLC-ESI-MS, IC-MS, or IC-ICP-MS [118–120], to separate and determine particular forms of the element. The literature data concerning arsenic speciation analytics with various hyphenated techniques is given in Table 1.

## 3. Antimony

Antimony, correspondingly to arsenic, belongs to group 15 of the periodic table. The physical and chemical qualities of both elements are similar. In the past, these two elements and their compounds were often determined together [134]. Its compounds were used as cosmetics in the Ancient Egypt. The alchemists used to believe that where there was antimony, there was gold as well. Antimony is a blue-white brittle metalloid. It behaves like a metal in most reaction. However, in some reactions, it demonstrates nonmetal characteristics. It has a rare quality—its solid form is less dense than its liquid one (similarly to water). 

Antimony occurs in coal seams (especially brown coal ones), diesel fuel, and gasoline. Its concentration in coal seams can be as high as 30 mg/kg and it can reach 100 mg/kg in ashes. On the other hand, its concentration in petroleum ranges between 0.001 and 0.1 mg/kg [111]. Antimony is a constituent of many alloys. It is also employed to produce fire retardants. Its compounds are used in medicine, and also in matches manufacturing, rubber vulcanization, china and ammunition production, as well as biomedicine, in which they are used as antiprotozoal agents and for treating tropical diseases. Antimony may occur at four oxidation states, that is, −3, +3, +4, and +5. It occurs mainly in Sb^3+^ and Sb^5+^ forms in the biological and geochemical environment. It is present in all units of the environment and its natural background in various environmental matrices is highly diversified [135]. Its content does not usually exceed 1 *μ*g/L in clean water and 500 mg/kg in rocks. Beside its natural sources, antimony also occurs as anthropogenic pollution. More than 20,000 tonnes of this element are exploited in Japan every year, whereas, interestingly, only 100 tonnes of arsenic, which is far more toxic, are used.

Antimony speciation is especially important in environmental and clinical analytics, as it is a toxic element whose bioavailability and reactivity depend not only on the oxidation state but also on the character of its particular compounds. In general, inorganic antimony compounds are more toxic than the organic ones. Sb(III) compounds are ten times more toxic than Sb(V) ones. On the other hand, the toxicity of antimony compounds is approximately ten times lesser than arsenic ones but it depends on their oxidation states and structure. Antimony in the elemental form is more toxic than its salts [14].

The biological role of antimony has not been fully understood yet. IARC (The International Agency for Research on Cancer) has concluded that there is enough evidence obtained from animal testing to recognize Sb_2_O_3_ as a carcinogenic compound [136]. However, the U.S. Environmental Protection Agency [137] and German Research Community [138] categorize antimony as a main pollutant but do not indicate its carcinogenicity.

The first works relating to antimony speciation were published in the early 1980s [139]. The speciation analytics of antimony and its compounds can be divided into determination of Sb(III) and Sb(V) and organic antimony compounds. The speciation is usually performed with gas or liquid chromatography. Capillary electrophoresis methods are also used [140]. What is important in the application of gas chromatography in speciation analytics of antimony and its compounds is the fact that all of its organic compounds are gases at room temperature. Unfortunately, they are very often unstable. Sb(III) and Sb(V) compounds are reduced to SbH_3_, which at room temperature is a toxic gas with garlic odor.

The speciation is carried out at two stages. First, the part of the sample undergoes reduction in the presence of high pH (without concentration). In these conditions, only Sb(III) compounds are reduced. Then, the other part of the sample is reduced and the amount of Sb(V) is calculated on the basis of the difference.

Organic antimony compounds determined in the environment include, among others, monomethylated methylstibonic amid [MeSbO(OH)_2_], dimethylated dimethylstibinic amid [Me_2_SbOOH], monomethylstibine [MeSbH_2_], and dimethylstibine [Me_2_SbH] [2]. Unfortunately, they are not available in the standard form and thus are obtained for the analytical needs in laboratories. Methylated Sb(V) compounds are usually reduced to suitable Sb(III) compounds, that is, MeSbO(OH)_2_ to MeSbH_2_, Me_2_SbOOH to Me_2_SbH, and Me_3_SbX_2_ to Me_3_Sb.

The most significant limitation related to separation methods based on gas chromatography is the fact that only the compounds undergoing the reduction process can be determined. Another problem is that Sb(III) is unstable and it easily oxidizes to Sb(V), hence many research papers indicate relatively high Sb(V) content in the analyzed samples.

Various forms of liquid chromatography are a possible alternative as they allow simultaneous determination of Sb(III) and Sb(V) compounds. Analytes such as Sb(III), Sb(V), or Me_3_SbX_2_, are anions themselves and can be determined as ions.

The most important problems with respect to speciation analytics of antimony and its compounds encompass the following.

- Standards of antimony compounds are unavailable and unstable. Sb(III) and Sb(V) standards are unstable. There are no certified reference materials for antimony compounds and suitable standards are difficult to obtain in chemical reactions. Very few inorganic and gaseous Me_3_Sb compounds are commercially available. Only trimethylated pentavalent antimony species (Me_3_SbCl_2_, MeSbBr_2_, Me_3_SbO, Me_3_Sb(OH)_2_) are produced with suitable purity and sufficient amount.
- There appear problems with low and ultra-low analyte contents in the samples, especially those with complex matrices [2].
- There exist problems related to unsuitable peak resolution and detection. Peak tailing, especially in the case of Sb(III) peak, appears while using liquid chromatography. Sb(III) forms divalent (and sometimes trivalent) ions in water solutions that strongly react with the resin. Besides, Sb(III), Sb(V) and Me_3_SbX_2_ are similar and difficult to separate.

Antimony and its compounds are most often determined in various types of water, such as drinking, mineral, and surface water. The information on the content of different speciation forms of antimony is not ample. It usually concerns specific water bodies and rivers or soil and plants growing in particular areas. Analyses concerning antimony and its compounds contents in biomedical samples attract great attention. Analysts find the matrix of such samples (urine, living tissues) challenging. They contain a lot of proteins, macromolecular substances, and enzymes that can bind with antimony compounds and cause its oxidation or reduction. The toxic activity mechanism of these compounds has not been fully understood yet and they are employed in treatment of various diseases, including the tropical ones. The literature data concerning antimony speciation analytics with various hyphenated techniques is given in Table 2.

## 4. Thallium

Thallium is a nonmetal from group 16 of the periodic table. It is a soft and silverish metal. It resembles lead and its surface quickly discolors due to oxidation processes when it is left in the air. It reacts with dilute strong inorganic acids (with the exception of hydrochloric acid) and displaces hydrogen out of them.

Thallium occurs at oxidation states of +1 and +3 in compounds. Tl^+^ cations are colorless and thallium(I) hydroxide is a soluble strong base. Tl^3+^ ions can exist in a solution only if its pH is close to 0. When it is higher, Tl(OH)_3_ precipitates [141]. Thallium compounds are highly toxic. The element is also toxic in the dust form as it oxidizes in the contact with air. Food and respiratory thallium poisonings are possible. One of the characteristic poisoning symptoms is hair loss preceded by hair follicle atrophy. Other signs include digestion disorders, pain, neuropsychiatric complications, as well as cardiovascular system damages [2]. In the past, thallium salts were often added to rodenticides.

The anthrophogenic environmental occurrence of thallium is caused by ore smelting, handling, and processing of waste products from the metallurgic industry [142]. Also, vegetation damages have been observed and researched around a cement plant using thallium-containing pyrite smelting residues [143]. Thallium occurs in the Earth crust and its mean amount equals to 0.6 mg/kg. It is also found, as a trace element, in ore seams containing sulfur and potassium compounds. Thallium(I) salts are easily absorbed through the skin and they usually enter living organisms in this way. Food is one of the important sources of thallium and its compounds. Consequently, food control monitoring is essential. Therefore, it is very important to determine sources of thallium in food and to investigate dietary intakes of this element. Thallium is widely distributed in the vegetable kingdom and is rapidly taken up by the plant root. Thus, the thallium-transfer from soils to plants should be investigated with respect to the correlation between the chemical form and the plant uptake of this element.

Thallium and its compounds are determined in urine, saliva, tissue, and blood samples in clinical analyses [144]. Thallium is a cumulative poison of high toxicity and is without taste, smell, and other warning attributes. Thallium and its compounds can enter the organism of persons working with them through the respiratory organs, through the gastrointestinal tract and through the skin. Industrial thallium poisoning can either be acute or chronic, but in all cases it is characterized by a long duration and severity of its course [142].

All results demonstrate the importance and the needs for more thallium speciation studies in different matrices, in order to obtain fundamental data for the treatment of subjects who suffer from chronic or acute thallium intoxication.

The following analytical techniques are used in the thallium analytics: atomic absorption spectrometry, coulometry, spectrophotometry, ICP-MS, laser inducted fluorescence spectrometry, or differential pulse stripping voltamperometry.

There are well-developed and described methods of antimony and arsenic analysis. On the other hand, there is a great pressure, both for the analytical chemists and toxicologists, to develop credible methodologies of thallium determination, especially in complex matrix samples. Determination of thallium and its compounds in water, snow, soil, sediments, or cement has been described in recent years [72]. Literature data concerning speciation analytics of thallium performed with various hyphenated techniques is presented in Table 3.

## 5. Summary and Conclusions

Arsenic, antimony, and thallium belong to a group of elements that, due to their physical, chemical and toxicological qualities, are particularly interesting objects of research within the framework of speciation analytics. The new approach towards the presence and role of these elements and their compounds is influenced by the constant development of analytical methods (including hyphenated techniques), toxicology, biochemistry, and environmental chemistry.

Most literature data concerns arsenic and its speciation forms whereas the least information relates to thallium and its compounds. New data connected with both the characteristics of selected speciation forms of these elements and methods enabling to determine them at lower concentration levels (in the complex matrix samples, such as food ones or living tissues) ought to emerge in the years to follow.

The hyphenated techniques, in which separation techniques are coupled with diverse selective and sensitive detection methods, are widely used in the speciation analytics of arsenic, antimony, and thallium. The hyphenated techniques create new and ever greater possibilities. Their main advantages include extremely low limits of detection and quantification, insignificant influence of interferences on the determination process, as well as very high precision and repeatability of determinations. Obviously, the hyphenated techniques pose certain limitations that involve the complexity and high price of the apparatus. As a result, they are not readily available and used in laboratories. What is more, the application of the hyphenated techniques requires excellent comprehension of analytical methodologies and the apparatus. These systems are very expensive and employed in scientific research rather than in routine analyses. Nevertheless, the hyphenated techniques have been constantly developing and gaining more and more importance, which is corroborated by the rising number of works pertaining to the subject.

## Figures and Tables

**Table 1:** Selected examples of application of hyphenated techniques in arsenic speciation.

Analytes	Analytical column	Mobile phase	Method of separation and detection	Matrix	Reference
As^3+^, As^5+^, MMA, DMA	Hamilton PRP-X100 Dionex AS7, AG7	75 mM Na_3_PO_4_, 2.5–50 mM HNO_3_	HPLC-ICP-MS	Surface water, mining water, underground water	[22]
As^3+^, As^5+^, MMA, DMA, AB, AC	Waters IC-Pak CM/D Waters Guard-Pak CM/D	NaHCO_3_/Na_2_CO_3_, HNO_3_	HPLC-ICP-MS	Water	[23]
As^3+^, As^5+^, MMA, DMA	Hamilton PRP-X100	10–200 mM NH_4_H_2_PO_4_	HPLC-ICP-DRC-MS	Sediments	[24]
As^3+^, As^5+^, MMA, DMA	Hamilton PRP-X100	30 mM NH_4_H_2_PO_4_	HPLC-ICP-MS	Soils	[25]
As^3+^, As^5+^, MMA, DMA, AB	Hamilton PRP-X100	20 mM NH_4_H_2_PO_4_	HPLC-ICP-DRC-MS	Polluted waters, leach	[26]
As^3+^, As^5+^, MMA, DMA, AB	Dionex AG-11, AS-11	NaOH, HNO_3_	HPLC-ICP-MS	Urine	[27]
As^3+^, As^5+^, MMA, DMA, AB, AC	Hamilton PRP-X100	0.3% HNO_3_, 10% methanol	HPLC-ICP-MS, HPLC-ESI MS	Fish sauce	[28]
As^3+^, As^5+^	Hamilton PRP X-100	Na_2_CO_3_	HPLC-ICP-MS	Surface water	[29]
As^3+^, As^5+^, MMA, DMA	Dionex AS7	HNO_3_	HPLC- ICP-MS, HPLC- INAA	Waters, rice extracts	[30]
As^3+^, As^5+^	Dionex AS9	NaOH, Na_2_CO_3_, NaHCO_3_	HPLC-SF-ICP-MS	Soils	[31]
As^3+^, As^5+^	Wescan Anion-S C18	EDTA	HPLC- ICP-MS	River waters, sludge	[32]
As^3+^, As^5+^	Biosil 125 SEC	CH_3_COONH_4_	HPLC- ICP-MS	Fish tissues	[33]
As^3+^, As^5+^	Waters IC-Pak A HC	NaOH, KNO_3_	HPLC- ICP-MS	Water, sludge	[34]
As^3+^, As^5+^, MMA, DMA, AB, AC	Develosil C30-UG-5, Chemcosorb 7SAX	sodium butanesulfonate, malonic acid, tetramethylammonium hydroxide, methanol, ammonium tartrate	HPLC- ICP-MS	Biological and environmental samples	[35]
As^3+^, As^5+^, MMA, DMA, AB	Dionex AG7, AS7	nitric acid, 1-benzene-2-disulfonic acid dipotassium salt	HPLC-ICP-MS	Seafood	[36]
As^3+^, As^5+^, MMA, DMA	Hamilton PRP-X100	NH_4_H_2_PO_4_, NH_4_HPO_4_, CH_3_COONH_4_, NaHCO_3_, NH_4_NO_3_	HPLC-ICP-MS	Soils, plant tissues	[37]
As^3+^, As^5+^	Dionex AG12A/AS 12A	disodium carbonate, sodium hydroxide, methanol	HPLC-ICP-MS	Iron rich water samples	[38]
As^3+^, As^5+^, MMA, DMA	Hamilton PRP X100	NH_4_NO_3_	HPLC-ICP-MS	Water	[39]
As^3+^, As^5+^, MMA, DMA	Hamilton PRP X100,	NH_4_H_2_PO_4_	HPLC- DF-ICP-MS	Cucumber sap	[40]
As^3+^, As^5+^, MMA, DMA, AB	Hamilton PRP X100	NH_4_H_2_PO_4_, NH_4_HPO_4_, MeOH	HPLC-ICP-MS	Rice, soil, straw, hair, nails	[41]
As^3+^, As^5+^, MMA, DMA, AB, AC, TMAO	Hamilton PRP X100, Zorbax 300-SCX	pyridine, NH_4_H_2_PO_4_	HPLC-SF-MS	Water, sediments, plants	[42]
As^3+^, As^5+^, DMA	G 3154A/101	EDTA, NH_4_H_2_PO_4_	HPLC-ICP-MS	Soils	[43]
As^3+^, As^5+^, MMA, DMA, AB	65001 and 65002	Na_2_EDTA, NH_4_H_2_PO_4_	HPLC-ICP-MS	Urine	[44]
As^3+^, As^5+^,MMA, DMA, AB	Supelcosil LC-SCX	pyridine, NaHCO_3_, Na_2_CO_3_	HPLC-ICP-MS HPLC-ES-MS	Hair, nail	[45]
As^3+^, As^5+^, MMA, DMA, AB, AC, TMAO	Dionex AG7, AS7	HNO_3_, MeOH	HPLC-ICP-MS	Seafood	[46]
As^3+^, As^5+^, MMA, DMA, AB	Dionex AS14, AS16, AS7	NaOH, NH_4_H_2_PO_4_	HPLC-ICP-MS	Poultry wastes	[47]
As^3+^, As^5+^, MMA, DMA, AB	Shodex Asahipak ES-502 N 7C*a *	HNO_3_, MeOH	HPLC-ICP-MS	Biological samples	[48]
As^3+^, As^5+^, MMA, DMA, AB	Hamilton PRP X100	(NH_4_)_2_SO_4_, (NH_4_)_3_PO_4_, NH_4_HCO_3_	HPLC-ICP-MS	Urine	[49]
As^3+^, As^5+^, MMA, DMA	Hamilton PRP X100	MeOH, NH_4_H_2_PO_4_	HPLC-ICP-MS	Peanut butter	[50]
As^3+^, As^5+^, MMA, DMA	Hamilton PRP X100	(NH_4_)_2_HPO_4_, NH_4_H_2_PO_4_	HPLC-ICP-MS	Wool	[51]
As^3+^, As^5+^, MMA, DMA, AC, AB, TMAO, TMAs	Hamilton PRP X100	NH_4_HPO_4_, (NH_4_)_2_HCO_3_, pyridine, MeOH	HPLC-ICP-MS, HPLC-ES-MS	Chinese seaweeds	[52]
As^3+^, As^5+^, MMA, DMA, AB	Hamilton PRP X100	NH_4_HPO_4_, NH_4_H_2_PO_4_	HPLC-ICP-MS	Biological samples (fish, rice, chicken)	[53]
As^3+^, As^5+^, MMA, DMA, AB	Dionex AS7	NH_4_H_2_PO_4_, NH_4_OH	HPLC-ICP-MS	Waters	[54]
As^3+^, As^5+^, MMA, DMA, AB, AC, TMAO, TMAI	Excelpak CHA-E11	HNO_3_	HPLC-ICP-MS	Rats urine	[55]
As^3+^, As^5+^, MMA, DMA, AB, AC	Dionex AS7, AG7	NaHCO_3_, Na_2_CO_3_	HPLC-ICP-MS	Fish, mussel extracts	[56]
As^3+^, As^5+^, MMA, DMA, AB, AC, TMAO, TeMAs^+^	Dionex AS7, AG7	HNO_3_, benzene-1,2-disulfonic acid	HPLC-ICP-MS	Fish oil	[57]
As^3+^, As^5+^, MMA, DMA, AC, AB TMAO, TeMAs^+^	Dionex AG4, AS4A	HNO_3_	HPLC-ICP-MS	Marine samples	[58]
As^3+^, As^5+^, MMA, DMA	Hamilton PRP X100	(NH_4_)_2_HPO_4_, MeOH	HPLC-ICP-MS	Soil extracts	[59]
As^3+^, As^5+^	Hamilton PRP X100	CH_3_COOH, NH_4_NO_3_, EDTA	HPLC-ICP-MS	Drinking water	[60]
As^3+^, As^5+^	Dionex AS12	NaHCO_3_, Na_2_CO_3_	HPLC-HGAAS	Mine tailings	[61]
As^3+^, As^5+^, MMA, DMA	—	—	FIA-HGAAS	Seawater	[62]
As^3+^, Total As	—	0.029 M HNO_3_, 0.024 M HCl	FIA-HGAAS	Water samples	[63]
As^3+^, As^5+^	—	—	FI-EHG-AAS	Synthetic samples	[64]
As^3+^, As^5+^, MMA, DMA	Hamilton PRP-X100	12 mM (NH_4_)_2_HPO_4_, 7.5 mM (NH_4_)_2_SO_4_ 10 mM (NH_4_)_2_CO_3_	HPLC-ICP-AES	Synthetic samples	[65]
As^3+^, As^5+^, MMA, DMA, AC, AB	Hamilton PRP-X100	NH_4_H_2_PO_4_, (NH_4_)_2_HPO_4_	HPLC-NAA	Water samples	[66]
As^3+^, As^5+^, MMA, DMA	LiChrospher 100 RP-18e adsorbent	tetrabutylammonium hydrogen sulfate	HPLC-ETAAS	Water samples	[67]
As^3+^, As^5+^, MMA, DMA	Hamilton PRP X-100	2,5 mM NaH_2_PO_4_, 2,5 mM Na_2_HPO_4_	HPLC-HGAAS	Natural waters	[68]
As^3+^, As^5+^, MMA, DMA	Alltech All-guard Adsorbosphere SAX 5 *μ*m	10 mM KH_2_PO_4_	HPLC-HG-AFS	Particulate matter	[69]
As^3+^, As^5+^, MMA, DMA	Dionex AS11, AG11	10 mM–100 mM NaOH	HPLC-HG-AFS	Polluted soil	[70]
As^3+^, As^5+^, MMA, DMA, AC, AB	Spherisorb ODS/NH_2_	5 mM NaH_2_PO_4_, 5 mM Na_2_HPO_4_,	HPLC-MO-HG-AAS, HPLC-ICP-MS	Water, urine	[71]

**Table 2:** Selected examples of application of hyphenated techniques in antimony speciation.

Analytes	Analytical column	Mobile phase	Method of separation and detection	Matrix	Reference
Sb^3+^, Sb^5+^	Hamilton PRP-X100	15 mM HNO_3_	HPLC- ICP-MS	Plants	[74]
Sb^3+^, Sb^5+^	Develosil C30-UG-5, Chemcosorb 7SAX	Malonic acid, sodium, 1-butylosulfonian, ammonium citrate, methanol	HPLC- ICP-MS	Biological and environmental samples	[35]
Sb^3+^, Sb^5+^, TMSbCl_2_	Hamilton PRP-X100, Dionex AS 14, AG 14	20 mM EDTA	HPLC- ICP-MS	Urine	[75, 76]
Sb^3+^, Sb^5+^	Hamilton PRP-X100	Phthalic acid, EDTA	HPLC- ICP-MS	Soil	[77, 78]
Sb^3+^, Sb^5+^	Hamilton PRP-X100	10 mM EDTA, 1 mM phthalic acid	HPLC- ICP-MS	Airborne particulate matter	[79, 80]
Sb^3+^, Sb^5+^, TMSbCl_2_, TMS(OH)_2_	Hamilton PRP-X100, Asahipak GS520HG	10 mM TMAH	HPLC-ICP-MS	Airborne particulate matter	[81, 82]
Sb^3+^, Sb^5+^	Hamilton PRP-X100	10 mM EDTA, 1 mM phthalic acid	HPLC-ICP-MS	Soils	[77, 78]
Sb^3+^, Sb^5+^, TMSbCl_2_	Hamilton PRP X-100	50 mM diammonium tartrate, 20 mM KOH	IC-HG-AFS, HPLC-ICP-MS	Plant extracts	[83]
Sb^3+^, Sb^5+^	Silica-based solid-phase extraction cartridges	Ammonium pyrrolidine dithiocarbamate	SPE-ICP-MS	Water	[84]
Sb^3+^, Sb^5+^	Hamilton PRP X-100	20 mM EDTA	HPLC-ICP-MS	Soil	[85]
Sb^3+^, Sb^5+^	Hamilton PRP X-100	10 mM EDTA, 1 mM phthalic acid	HPLC-ICP-MS	Thirteen fractions of airborne particulate matter	[86]
Sb^3+^, Sb^5+^,	Dimercaptosuccinic acid chemically modifying mesoporous titanium dioxide microcolumn	Water	SPE-ICP-OES	Natural waters	[87]
Sb^3+^, Sb^5+^, TMSbCl_2_, TMS(OH)_2_	Hamilton PRP-X100, Hamilton PRPX-200, Supelcosil LC-SCX, Hamilton PRP1, Phenomenex Intersil 5 ODS	KH_2_PO_4_/K_2_HPO_4_ 0.5–5 mM, KHCO_3_/K_2_CO_3_ 1–50 mM, pyridine, 2,6 dicarboxylic acid (PDCA): 5–20 mM EDTA, 5–50 mM HNO_3_, 1–4 mM, ethylenediamine	HPLC-ICP-MS, HPLC-FAAS	Environmental samples	[88]
Sb^3+^, Sb^5+^	Dionex AS14	2 mM NH_4_HCO_3_, 2.2–45 mM tartaric acid	HPLC-ICP-MS	Fish extracts	[89]
Sb^3+^, Sb^5+^	—	Chloranilic acid (2,5-dichloro-3,6-dihy-droxy-1,4-benzoquinone)	HMDE	Water	[90]
Sb^3+^, Sb^5+^, MMSb^4+^, DMSb^3+^, TMSb^2+^	Teflon tube packed with SE-30 on Chromosorb W-HP	Liquid nitrogen	HG-SPE-ICP-MS	Surface waters	[91]
Sb^3+^, Sb^5+^	—	40 mM thioglycolic acid	SPE-ICP-OES, SPE-ICP-MS	Water, yeast extract	[92]
Sb^3+^, Sb^5+^	Hamilton PRP-X100	20 mM EDTA, 2 mM KHP	HPLC-ICP-MS	Waters	[93]
Sb^3+^, Total Sb	—	l-cysteine, hydrochloric acid	HG -AFS	Herbs	[94]
Sb^3+^, Sb^5+^	—	H_2_O, 0.05 M EDTA, 0.25 M H_2_SO_4_	HG -AFS	Soil	[95]
Sb^3+^, Sb^5+^	Hamilton PRP-X100	10 mM EDTA, 1 mM phthalic acid	HPLC-ICP-MS	Moat water	[79, 80]
Sb^3+^, Sb^5+^	Hamilton PRP-X100	20 mM EDTA, 2 mM KHP	HPLC-ICP-MS	Citrus juices Mineral water	[96]
Sb^3+^, Sb^5+^	Hamilton PRP-X100	5 mM HNO_3_	HPLC-ICP-MS	Cell extracts	[74]
Sb^3+^, Sb^5+^, TMSbCl_2_	Hamilton PRP-X100	20 mM EDTA, 2 mM KHP	HPLC-HG-AFS	Sea water,	[97]
Sb^3+^, Sb^5+^,	Hamilton PRP-X100	EDTA 2–20 mM	HPLC-ICP-MS, FI-HG-ICP-MS	Biological samples	[98]
Sb^3+^, Sb^5+^, TMSbCl_2_	Dionex AS15/AG15	20 mM EDTA, NH_4_OH, 1 mM EDTA	HPLC-ICP-MS	*Pteris vittata* samples	
Sb^3+^, Sb^5+^	Microcolumn with immobilized aminoacid	HCl	HG-ICP-OES	Urine	[99]
Sb^3+^, Sb^5+^	Hamilton PRP-X100	200 mM diammonium tartrate	HPLC-HG-AFS	Airborne particulate matter	[100]
Sb^3+^, Sb^5+^, TMSbCl_2_	Hamilton PRP-X100	20 mM EDTA + 2 mM potassium hydrogen phthalate, 50 mM diammonium hydrogen phosphate	HPLC-(UV)-HG-AFS	Algae and mollusk extracts	[101]
Sb^3+^, Sb^5+^, TMSbO	Hamilton PRP-X100	2 mM phthalic acid 2 mM 4-hydroxybenzoic acid	HPLC-ICP-MS, HPLC-ICP-OES	Surface water, soil extracts	[102]
Sb^3+^, Sb^5+^, TMSbCl_2_	Hamilton PRP-X100	EDTA-K_2_HPO_4 _20 mM	HPLC-HG-AFS	Sediments	[103]
Sb^3+^, Sb^5+^, TMSbCl_2_	Hamilton PRP-X100	20 mM EDTA, 8 mM KHP 1 mM K_2_CO_3_	HPLC-HG-AFS	Environmental samples	[104]
Sb^3+^, Sb^5+^	Hamilton PRP-X100	250 mM diammonium tartrate, 20 mM KOH	HPLC-ICP-MS	Volcanic ash	[105]
Sb^3+^, Sb^5+^	—	1.5 M HAc, 0.5 M H_2_SO_4_	FIA-HG-AAS	Biological samples	[106]
Sb^3+^, Sb^5+^, TMSbCl_2_	CETAC ION-120 Dionex AS 14	2 mM NH_4_HCO_3_, 1 mM tartaric acid, 1.25 mM EDTA	HPLC-ICP-MS	Tap water	[75, 76]
Sb^3+^, Sb^5+^	—	5.0∗10^−5 ^M BPHA, Triton X-114 (0.20% (v=v))	SPE-FAAS	Water	[107]
Sb^3+^, Sb^5+^, TMSbCl_2_	Hamilton PRP-X100	20 mM EDTA, 2 mM KHP, 50 mM (NH_4_)_2_HPO_4_	HPLC-HG-AFS	Sea water	[97]
Sb^3+^, Total Sb	—	—	HS-SDME-ETAAS	Lake water, soils	[108]
Sb^3+^, Sb^5+^, TMSbCl_2_	Hamilton PRP-X100, Dionex AS4A-SC	12 mM tetra-methylammonium hydroxide 3 mM tetra-methylammonium hydroxide	HPLC-ICP-MS	Environmental samples	[109]
Sb^3+^, Sb^5+^	Dionex AS 14, AG 14	2 mM ammonium hydrogen carbonate, 2.2 mM tartaric acid, 2 mM ammonium hydrogen carbonate, 45 mM tartaric acid,	HPLC-ICP-MS	Synthetic samples	[110]
Sb^3+^, Sb^5+^	Hamilton PRP-X100	250 mM diammonium tartrate, 20 mM KOH	HPLC-HG-AFS, HPLC-ICP-MS	Coal fly ash	[111]
Sb^3+^, Sb^5+^	Synchropak Q300	5 mM EDTA, 2 mM phthalic acid	HPLC-ICP-MS	Tap water	[81, 82]
Sb^3+^, Sb^5+^	Hamilton PRP-X100	20 mM EDTA, 2 mM phthalic acid	HPLC-ICP-MS, HPLC-ESI-MS	Yoghurt, juice, urine	[112]
Sb^3+^, Sb^5+^ TMSbCl_2_	Phenomenex SAX-SB	100 mM ammonium tartrate	HPLC-ICP-MS	Synthetic solutions	[113]
Sb^3+^, Sb^5+^, TMSbCl_2_	ION-120, Supelcosil SAX Hamilton PRP-X100, Dionex AS 14, Dionex AS 9	NH_4_HCO_3,_ EDTA, Tartaric acid	HPLC-HG-AAS	Environmental samples	[114]
Sb^3+^, Sb^5+^	Supelcosil LC-SAX 1	50 mM ammonium tetrate	HPLC-HG-AAS	Water	[115]
Sb^3+^, Sb^5+^	—	3 g/L L-cysteine	HG-ICP-AES	Spiked water samples	[116]

**Table 3:** Selected examples of application of hyphenated techniques in thalium speciation.

Analytes	Analytical column	Mobile phase	Method of separation and detection	Matrix	Reference
Tl^1+^, Tl^3+^	Chelex-100 resin column	14% HNO_3_	SPE-ICP-MS, SPE-GFAAS	River waters	[121]
Tl^1+^, Tl^3+^	L-Tyr-CNTs	10% HNO_3_	SPE-STPF-ETAAS	Tap waters	[122]
Tl^1+^, Tl^3+^	—	Extraction with 1-pyrrolidinecarbodithioic acid, APDC	Extraction-ETAAS	Wine	[123]
Tl^1+^, Tl^3+^	—	Triton X-114, sodium dodecyl sulfate, DTPA	Extraction-ICP-MS	Environmental water samples	[124]
Tl^1+^, Tl^3+^	Hamilton PRP-X100	100 mM ammonium acetate, and 5 mM DTPA	IC-ICP-MS	Plants extracts	[125]
Tl^1+^, Tl^3+^	SPE (Dowex 50-8X)	14% HNO_3_	SPE-ICP-MS	Lake waters	[126]
Tl^1+^, Tl^3+^	Microcolumn (sodium dodecyl sulfate + alumina)	1 M Na_2_S_2_O_3_	FI-FAAS	Water end wastewater	[127]
Tl^1+^, Tl^3+^	—	Hydrazine	FIA and spectrofluorimetric	Real samples	[128]
Tl^1+^, Tl^3+^	Chelex-100 resin column	3.2 M HNO_3_	SPE-ICP-MS	Lake waters	[129]
Tl^1+^, Tl^3+^	Microcolumn (multiwalled carbon nanotubes)	1 M HNO_3_	SPE-STPF-ETAAS	Water	[130]
Tl^1+^, Tl^3+^	Dionex CG12A	0.015 M HNO_3_	IC-ICP-OES, IC-ICP-MS	Water	[131]
Tl^1+^, Tl^3+^	Dionex AG12A CG12A	HNO_3_, HCl	IC-ICP-MS	Water	[132]
Me_2_Tl^+^	Microcolumn (filled with AG1-X8)	NaDDTC, HNO_3_, MIBK,	PTI-IDMS	Oceanic water	[133]

## Abbreviations

AB: Arsenobetaine
AC: Arsenecholine
BPHA: N-Benzoyl-N-phenyhydroxylamine
DMA: Dimethylarsinic Acid
EDTA: Ethylenediaminetetraacetic acid
FIA-HGAAS: Flow Injection Analysis coupled to Hydride Generation Atomic Absorption Spectrometry
FIA-HG-AAS: Flow Injection Analysis with Hydride Generation Atomic Absorption
FI-EHG-AAS: Flow Injection Analysis coupled to Electrochemical Hydride Generation Atomic Absorption Spectrometry
FI-HG-ICP-MS: Flow Injection Hydride Generation with Inductively Coupled Plasma Mass Spectrometry
HG-AFS: Hydride Generation coupled to Atomic Fluorescence Spectrometry
HG-ICP-AES: Hydride Generation Inductively coupled to Plasma Atomic Emission Spectrometry
HG-SPE-ICP-MS: Solid Phase Extraction Hydride Generation Atomic Fluorescence Spectrometry
HMDE: Hanging Mercury Drop Electrode
HPLC- INAA: High Performance Liquid Chromatography with Instrumental Neutron Activation Analysis
HPLC-DF-ICP-MS: High Performance Liquid Chromatography With Double Focusing Sector Field Inductively Coupled Plasma Mass Spectrometry
HPLC-ESI-MS: High Performance Liquid Chromatography with Electrospray Ionization Mass Spectrometry
HPLC-ETAAS: High Performance Liquid Chromatography with Electrothermal Atomic Absorption Spectrometry
HPLC-FAAS: High Performance Liquid Chromatography with Flame Atomic Absorption Spectrometry
HPLC-HGAAS: High Performance Liquid Chromatography coupled to Hydride Generation Atomic Absorption Spectrometry
HPLC-HG-AFS: High Performance Liquid Chromatography coupled to Hydride Generation Atomic Fluorescence Spectrometry
HPLC-ICP-AES: High Performance Liquid Chromatography Inductively coupled to Plasma Atomic Emission Spectroscopy
HPLC-ICP-DRC-MS: High Performance Liquid Chromatography with Inductively Coupled to Plasma Mass Spectrometry With Dynamic Reaction Cell
HPLC-ICP-MS: High Performance Liquid Chromatography with Inductively Coupled Plasma Mass Spectrometry
HPLC-MO-HG-AAS: High Performance Liquid Chromatography Microwave-Assisted Oven Coupled to Hydride Generation Atomic Absorption Spectrometry
HPLC-NAA: High Performance Liquid Chromatography coupled to Neutron Activation Analysis
HPLC-SF-ICP-MS: High Performance Liquid Chromatography with Sector Field Inductively Coupled Plasma Mass Spectrometry
IC-HG-AFS: Ion Chromatography Coupled to Hydride Generation Atomic Fluorescence Spectrometry
IC-ICP-MS: Ion Chromatography Coupled to Inductively Coupled Plasma Mass Spectrometry
IC-ICP-OES: Ion Chromatography coupled to Inductively coupled to Plasma Atomic Emission Spectroscopy
Me_2_Tl^+^: Dimethyl Thallium Ion
MIBK: Methyl Isobuthyl Ketone
MMA: Monomethylarsonic Acid
NaDDTC: Sodium Diethyldithiocarbamate
PDCA: 2,6 Dicarboxylic Acid
PTI-IDMS: Positive Thermal Ionization Isotope Dilution Mess Spectrometry
SPE-FAAS: Solid Phase Extraction with Flame Atomic Absorption Spectrometry
SPE-GFAAS: Solid Phase Extraction Graphite Furnace Atomic Absorption Spectrometry
SPE-ICP-MS: Solid Phase Extraction With Inductively Coupled Plasma Mass Spectrometry
SPE-ICP-OES: Solid Phase Extraction With Inductively Coupled Plasma Atomic Emission Spectroscopy
SPE-STPF-ETAAS: Solid Phase Extraction Stabilized Temperature Platform Furnace With Electrothermal Atomic Absorption Spectrometry
TeMAs^+^: Tertramethylarsonium Ion
TMAH: Tetramethylammonium hydroxide
TMAI: Tetramethylarsonium Iodide
TMAO: Trimethylarsine Oxide
TMSbCl_2_: Trimethylantimony Dichloride
TMSbO: Trimethylstiboxide.
